# Correlation of Posterior Tibial Tendon Ultrasound with Calcaneal Inclination Angle in Indonesian Professional Athletes with Medial Ankle Pain

**DOI:** 10.7150/ijms.98222

**Published:** 2024-07-16

**Authors:** Rosy Setiawati, Alfian Hasbi, Paulus Rahardjo, Damayanti Tinduh, Alit Pawana, Vincent Geraldus Enoch Lusida, Giuseppe Guglielmi, Suresh Mukherji

**Affiliations:** 1 Department of Radiology, Faculty of Medicine, Universitas Airlangga, Surabaya, Indonesia.; 2 Department of Physical Medicine and Rehabilitation, Faculty of Medicine, Universitas Airlangga, Surabaya, Indonesia.; 3 Faculty of Medicine, Universitas Airlangga, Surabaya, Indonesia.; 4 Department of Clinical and Experimental Medicine, Department of Radiology, School of Medicine, Foggia University, Foggia, Italy.; 5 Department of Radiology, Michigan State University, Michigan, United States of America.

**Keywords:** Posterior tibialis tendon dysfunction, adult acquired flatfoot deformity, ultrasound, Calcaneal inclination angle, tenosynovitis, tear, PTTD, AAFD

## Abstract

**Background:** Adult-acquired flatfoot deformity (AAFD) is characterized by partial or complete flattening of the longitudinal medial arch, which develops after maturity. AAFD secondary to posterior tibialis tendon dysfunction (PTTD) is one of professional athletes' most common foot and ankle pathologies. Different modalities and procedures can be used to establish the diagnosis of AAFD and PTTD. However, imaging measurements such as the calcaneal inclination index and ultrasonography (US) of the posterior tibialis tendon (PTT) in professional athletes with medial ankle and focal pain along the PTT have yet to be widely studied. This study investigates the correlation of PTT ultrasound for evaluating PTTD with calcaneal inclination angle (CIA) for evaluating AAFD in professional athletes with medial ankle and focal pain along the PTT. Through this study, clinicians and radiologists may benefit from considering AAFD in athletes with PTTD.

**Methods:** 112 Indonesian professional athletes with medial ankle or foot pain and focal pain along the direction of the PTT underwent foot radiography using the CIA and ankle ultrasound to observe PTT abnormalities.

**Results:** A negative correlation between fluid thickness surrounding the PTT and the CIA (p<0.001; 95% CI - 0.945, - 0.885), as well as a negative correlation between PTT thickness and CIA (p<0.001, 95% CI - 0.926, - 0.845), with a correlation coefficient (r) of - 0.921 and - 0.892, respectively. No significant correlation was found between PTT tear and CIA (p = 0.728; 95% CI -0.223, - 0.159; r - 0.033).

**Conclusion:** This study showed a negative correlation between PTTD and AAFD via ultrasound and CIA in professional athletes with medial ankle and focal pain along the PTT. A better understanding of PTTD and AAFD imaging will lead to more effective management and prompt treatment.

## Introduction

Human feet have become exceptionally specialized in performing two divergent functions: static balance and propulsion.^1^ Various arches form a series of bones that achieve balance, propulsion, and shock absorption throughout standing and the gait cycle.^2^, ^3^ Flatfoot occurs from a partial or complete collapse of the arch. Excessive pronation caused by flatfoot disrupts these functions, mainly during explosive forces in sprinting and long-distance running.^4^ Adult-acquired flatfoot deformity (AAFD) is characterized by partial or complete flattening of the longitudinal medial arch that develops after maturity.^5^ AAFD may cause some symptoms and complications, such as postural disturbance, knee pain, and foot pain, all of which are the most common causes of pain in professional athletes with sports injuries.^6^ Furthermore, AAFD may negatively affect athlete balance and vertical jump.^2^

AAFD, secondary to posterior tibialis tendon dysfunction (PTTD), is one of the most frequent foot and ankle pathologies in professional athletes and approximately 5 million adults in America.^7^ It is classified according to clinical symptoms, the severity of the foot deformity, and the flexibility of the deformity.^8^-^10^ The disorder is most commonly initiated by dysfunction of the posterior tibialis tendon (PTT), which generally maintains the talonavicular joint at the apex of the three arches of the foot. PTTD manifests as tendinosis, tenosynovitis, or tears. There are several causes of AAFD, including PTTD, trauma, neuropathy, neuromuscular disease, and inflammatory arthritis, the most common of which is PTTD.^5^, ^11^ When an individual has PTTD, the body weight distribution is redirected to the supporting structures, which consist of the spring ligament complex, sinus tarsi ligament, and deltoid ligament complex. The distribution of this structure can cause flatfoot deformities.^8^

Foot morphology is an essential intrinsic factor for athletic performance. From an anatomical and biomechanical point of view, minor alterations in foot and ankle structure or alignment are reflected in sports performance. Repeated movements in sports associated with muscle imbalances impact the incidence and development of certain postural disorders; therefore, they can affect foot biomechanics, reduce motor performance, and increase the risk of injury in competitive athletes.^12^ A study on the variability of foot arches among practitioners of different sports has already been carried out, which found that sports athletes have the most significant risk factor for AAFD and PTTD.^13^

The gold standard procedure for diagnosing AAFD is weight-bearing radiography.^14^ Radiographic measurements are necessary for anteroposterior, lateral, and hindfoot views. The lateral views will assess the angle of arch collapse, mainly by measuring the Calcaneal inclination angle (CIA) and lateral first tarsometatarsal angle.^15^ Compared to other measurement methods, CIA provides the best assessment of injury to the supporting structures of the medial longitudinal arch.^8^ Other structures apart from the PTT that significantly affect the medial longitudinal arch are the gastrocnemius and soleus muscle.^9^ Contracture of the achilles tendon deforms the alignment of the midfoot. However, flatfoot patients having done tendon achilles lengthening did not significantly associate with the diagnosis when measured using CIA, contrary to lateral talo-first metatarsal angle.^16^

Several studies have indicated that ultrasound may help assess the posterior tibial tendon compared to the more time-consuming and costly MRI. Results from the ultrasonographic evaluation of the posterior tibial tendon were equivalent to MRI in 87-94% of patients.^17^, ^18^ Ultrasound has recently become a widely used diagnostic tool for PT tendon lesions in sports injuries because of its ability to visualize fibers in the desired projection and real-time functional state, as well as a variety of PTTD findings, including tearing, tendon fiber thickening, heterogeneity, tendon sheath thickening, echogenicity, and fluid of the tendon sheath.^5^, ^19^, ^20^ Studies have stated that high-resolution ultrasound may be as sensitive and specific as contrast-enhanced MRI^21^, if not slightly more accurate, for detecting PTTD.^17^

However, imaging measurements, such as the CIA and ultrasonography (US) of PTT in professional athletes with medial ankle pain, have yet to be widely studied. This study investigates the correlation of posterior tibial tendon ultrasound with the CIA for evaluating PTTD in professional athletes with medial ankle and focal pain along the PTT. Through this study, clinicians and radiologists treating athletes with medial ankle and focal pain along the PTT may benefit from considering AAFD in patients with PTTD via these imaging measurements. Building upon previous studies, we hypothesize a negative impact between posterior tibial tendon ultrasound and CIA.

## Methods

### Subjects

This study was conducted in accordance with the Declaration of Helsinki and approved by the Institutional Review Board of the Medical Research Ethics Committee of Dr. Soetomo Academic General Hospital, Surabaya (2043/118/KEPK/III/2020). Upon admission, all participants, including those who are under 18 years old, provided written informed consent to participate in the study. All methods were performed in accordance with the relevant guidelines and regulations.

The sample consisted of 112 professional athletes. The inclusion criteria consisted of Indonesian athletes with medial ankle or foot pain and focal pain along the direction of the PTT; the age range was 15 to 20 years old. Patients with a history of ankle or pediatric trauma and previous intensive treatment for flatfoot were excluded from the study. The sampling method used was the total sampling of athletes participating in annual Indonesian sports competitions screened at the Sports Clinic of Dr. Soetomo General Academic Hospital Surabaya in March 2020. These professional athletes regularly participated in sports competitions and earned income from these activities. They minimally have two years of experience involved in national/international competitions. This observational analytical study using a cross-sectional design assessed the correlation between PTTD via US measurements of the PTT, including fluid thickness around the PTT, PTT sheath thickness, PTT tear, and AAFD via the CIA.

### Ultrasonography

**Figure 1:**
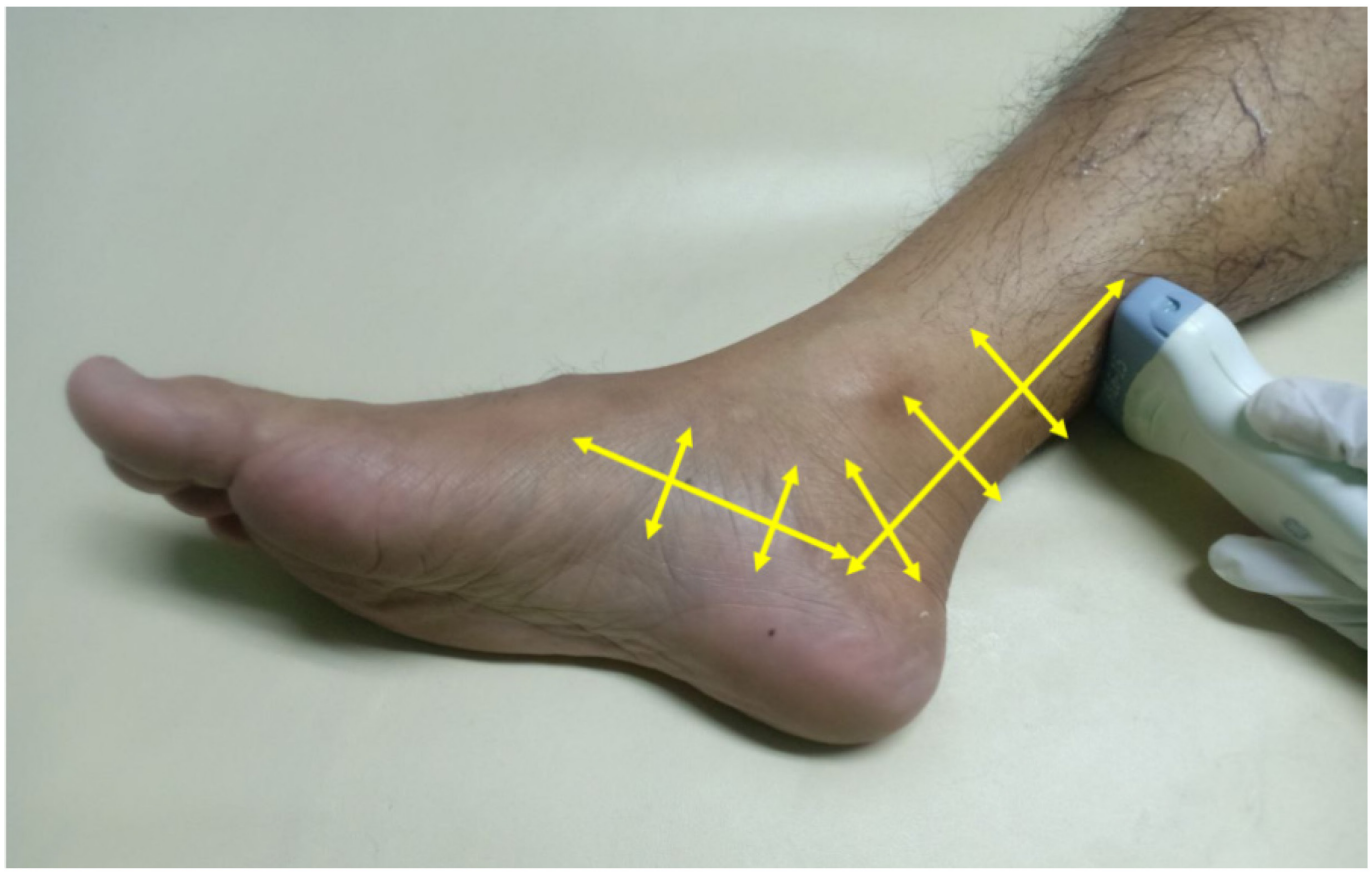
Transducer positioning for posterior tibial tendon ultrasound scanning positioning of lateral x-ray weight bearing foot.

**Figure 2:**
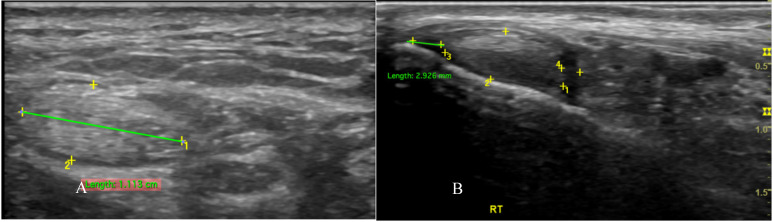
Transverse sonographic of posterior tibialis tendon with tenosynovitis showed thickening of the tendon fiber (A) with fluid in the tendon sheath (B).

**Figure 3:**
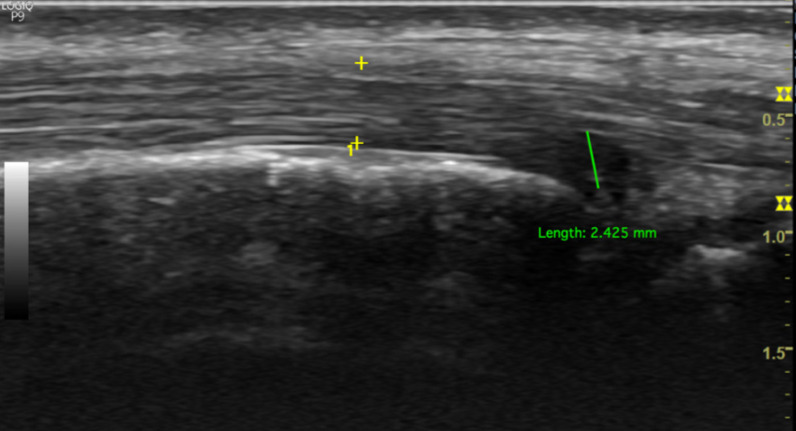
Longitudinal sonographic of posterior tibialis tendon with partial tear.

PTT is well-assessed using US. We used a GE Logiq P9 ultrasound machine with an 18 MHz linear array transducer to scan the PTT. The position of the transducer in the PTT for ultrasound scanning is essential for obtaining accurate results. The subjects were placed in the lateral decubitus position. The PTT was scanned in longitudinal and transverse positions (Figure 1). Posterior tibialis dysfunction appears as tenosynovitis, tears, or tendinosis. The free fluid collection that created a hypoechoic lesion around the PTT and a sheath size greater than 7mm was tenosynovitis (hyperechoic central structure with a hypoechoic halo) on the ultrasound transverse position (Figure 2).^5^, ^22^ A PTT tear or rupture appeared on ultrasound as an empty tibial groove with an intermuscular gap in the PTT (Figure 3). Tendinosis results in posterior tibial tendon thickening, with heterogeneous hypoechoic regions replacing the normal fibrillar architecture.^20^

### Radiography

**Figure 4:**
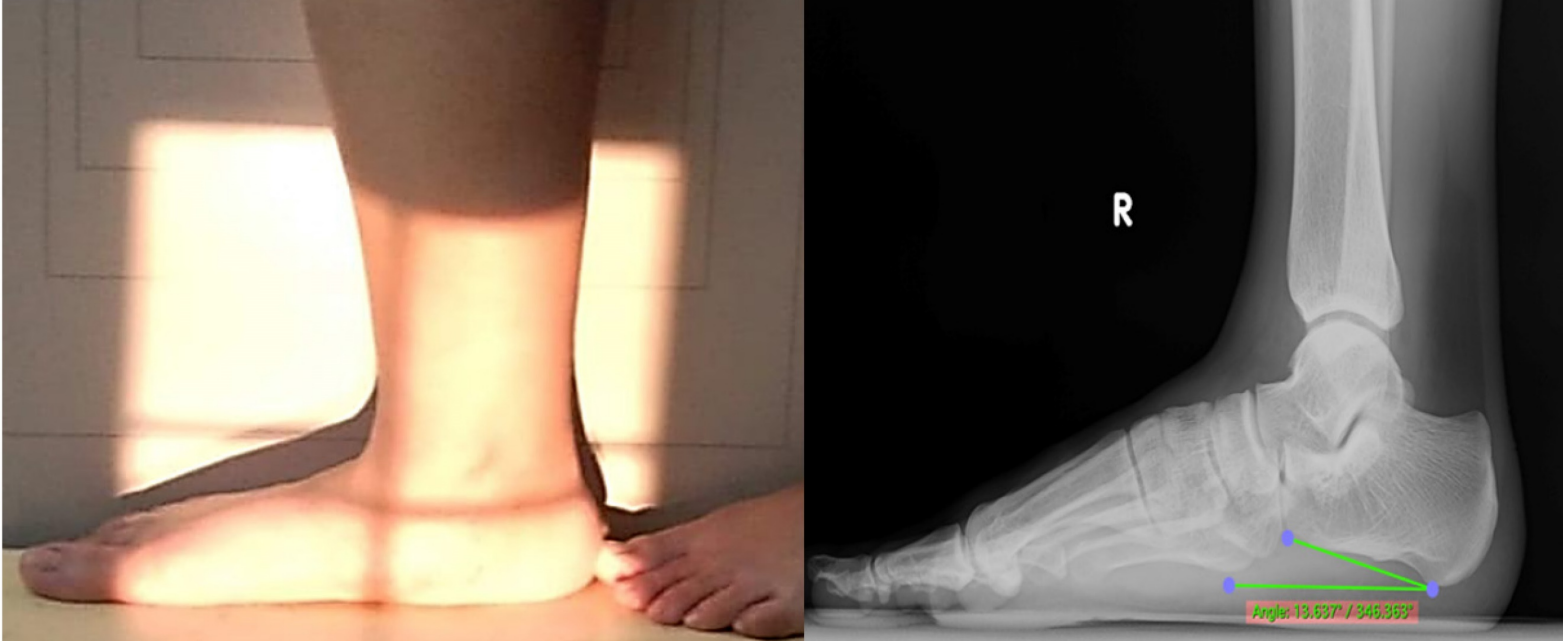
Positioning of lateral x-ray weight bearing foot.

**Figure 5:**
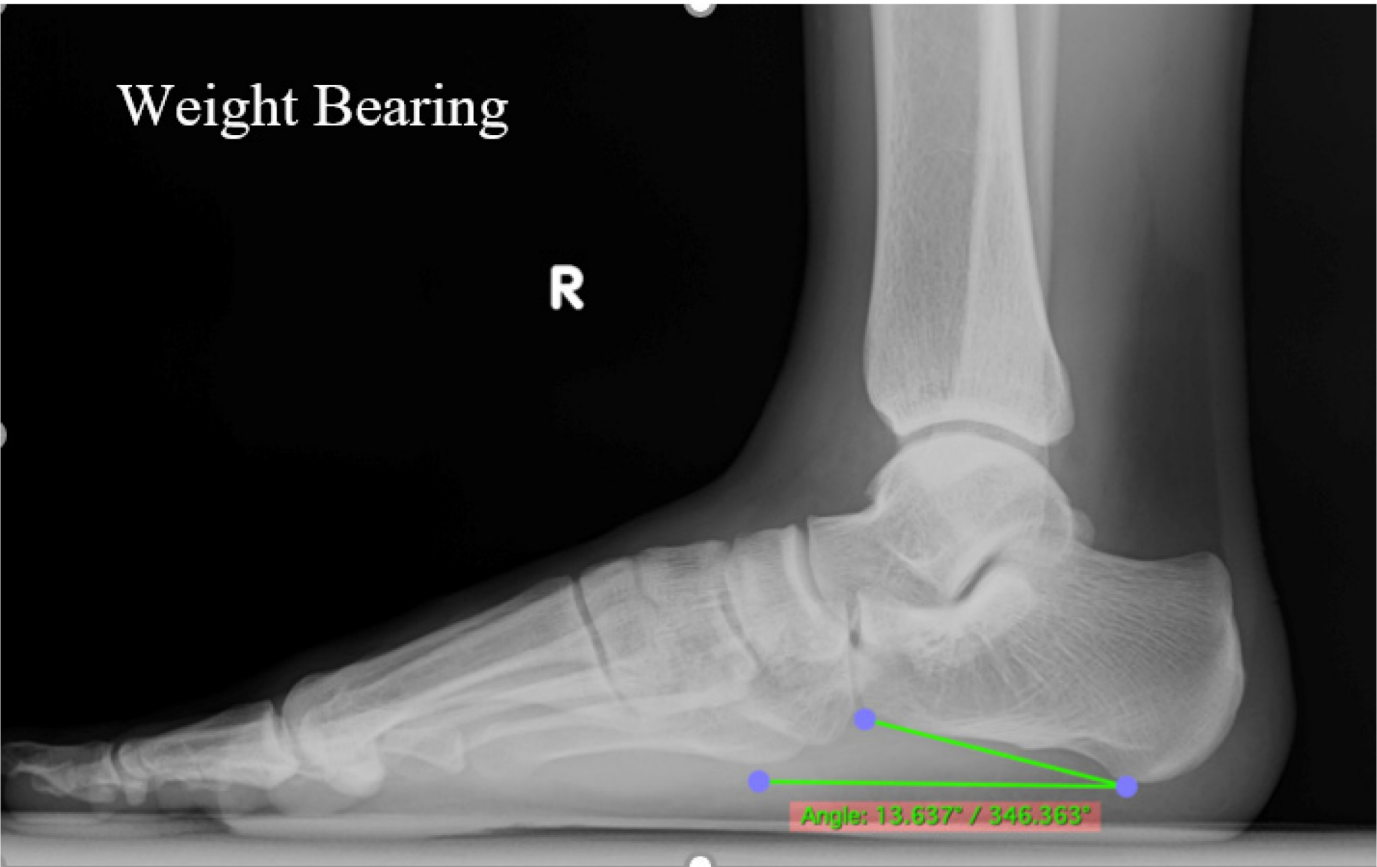
Lateral radiograph shows the calcaneal inclination angle.

Weight-bearing foot and ankle lateral projection radiographs remain the gold standard for diagnosing AAFD.^14^ The X-ray machine unit of the DRGEM Stationary X-ray System GXR-C52SD was used for the examination. Standing lateral views of the foot were obtained for all athletes (Figure 4). All radiographs were obtained in a weight-bearing position using a standardized technique with the same digital radiography system. The athletes were advised to let passive movement go through their ankle and foot arch to ensure no manipulations in the measurements. The digital X-ray detector (film) and X-ray tube (source) were 35-40 inches apart in both the lateral views of the foot. The CIA is one of the most common measurements to diagnose flatfoot deformity (Figure 5). The CIA was measured by the angle between the line, parallel to the plantar calcaneal surface, and the horizontal plane. The plantar calcaneal surface is measured by the most inferior portion of the calcaneal tuberosity and the most distal and inferior point of the calcaneus at the calcaneocuboid joint.^23^ Alignment angles (degrees) <18° were categorized as pes planus.^5^, ^24^, ^25^

A senior musculoskeletal radiology consultant with over ten years of experience performed all radiographic measurements and US examinations. Different clinicians measured the radiographic examination in separate rooms to determine inter-rater reliability, and the US radiologist was blinded to the radiographic results.

### Statistical Analysis

SPSS 24 software (IBM, Armonk, New York, United States) was used for statistical analysis. Data from US and radiographic radiography were arranged in a table and analyzed. All variables (free fluid collection in the tendon sheath, tendon thickness, tendon tear, and CIA) were tested for distribution using the Kolmogorov-Smirnov test. P > 0.05 was considered to be normally distributed.

The inter-rater reliability of US measurements was calculated using the intraclass correlation coefficient (ICC). The ICC classification is as follows: less than 0.5 was poor; 0.5-0.75 was moderate; 0.75-0.9 was good; and above 0.9 was excellent.^26^ The correlations between US measurements and CIA were assessed with Pearson correlation coefficients if the distribution is normal and Spearman's rank correlation if the distribution is not normal. The correlations are scaled from -1 to +1, where 0 indicates no linear relationship and -1 and +1 indicate an absolute negative and positive relationship, respectively.^27^. P < 0.05 was considered statistically significant with a confidence interval of 95%.

## Results

In this study of 112 samples, we have 42 male participants and 70 female participants between the ages of 15 and 20 years old. Wrestling (34) had the highest number of athletes, followed by fencing (26), handball (20), athletics (14), martial arts (14) and gymnastics (4). US findings in this study measured the tendon sheath fluid thickness around the PTT, thickness of the PTT sheath, presence of a tear in the PTT, and CIA. The inter-rater reliability results demonstrated that the ICC of ultrasound findings was good for tendon sheath fluid thickness (r = 0.89; CI 0.83, 0.94) and PTT sheath thickness (r = 0.87; CI 0.78, 0.92). We found the range of fluid thickness of the sample was 0-5.8 mm, with a mean value of 2.57 mm and a median value of 2.8 mm. The thickness of the PTT sheath ranges from 5.5 to 13 mm, with a mean value of 8.25 mm and a median value of 8.2 mm. PTT tears range from 1.2 to 2.7 mm with a mean value of 0.13 mm. The CIA from radiographic measurements ranges from 9.5-23º with a mean value of 17.2° and a median of 16.6° (**Table 1**). Of 112 samples, we found 67% had first-degree of flatfoot, 20% second flatfoot, and 2% had third-degree flatfoot. Radiographic examination showed similar results: approximately 70% were flatfoot (Table 1).

Comparing the total number of athletes in each type of sport (Table 2), it was found that the highest percentage of athletes with tenosynovitis were in gymnastics (100%), followed by wrestling (71.59%), and martial arts athletes had the lowest percentage of tenosynovitis (35.72%). The highest percentage of athletes with tendon tear is fencing (7.69%), followed by martial arts and athletics, and the lowest percentage was gymnastics (0%). The highest percentage of athletes in which flatfoot was discovered was in gymnastics (100%), followed by fencing (84.15%), and the lowest percentage in martial arts (50%).

Based on the Kolmogorov-Smirnov distribution test, it was found that the fluid thickness, tendon thickness, tendon tear, and CIA were not distributed normally. The correlation hypothesis test was carried out using Spearman's rank correlation test. Having done the test, a strong negative correlation was found between the fluid thickness surrounding the PTT and the CIA, with a correlation coefficient (r) of - 0.921 (p <0.001; 95% CI - 0.945, - 0.885). A strong negative correlation was also found between the PTT sheath thickness and CIA, with a correlation coefficient (r) of 0.892 (p<0.001; 95% CI - 0.926, - 0.845). However, the correlation between PTT tear and CIA was not significant, with a correlation coefficient (r) of 0.033 (p = 0.728; 95% CI - 0.223, - 0.159) (Table 3).

## Discussion

The current study's results indicate a statistically significant negative correlation between PTT sheath thickening and tendon sheath fluid, as diagnosed through ultrasound and CIA in professional athletes with medial ankle pain and pain along the PTT. Our results are supported by Karasick and Schweitzer, who evaluated patients with surgically proven PTT tears and found that 50% of the patients had a decreased calcaneal pitch angle, and also by Lin *et al.*, who showed a significant association between some radiographic measurements, including calcaneal inclination angle with PTTD diagnosed on MRI.^8^, ^28^ The angle of calcaneal inclination is associated with injuries to the supporting structures of the medial longitudinal arch. Bone landmarks used to calculate the calcaneal pitch angle are often preserved even in cases of severe leg deformity; therefore, precise measurements can still be obtained.^8^, ^29^ However, a study using similar variables found that PTT tendinosis and isolated tenosynovitis had a poor association with calcaneal inclination angle.^8^ Our study differs in sample characteristics, which may indicate that professional athletes are more susceptible to further chronic tendon injuries and overuse injuries, which cause tendon elongation or other anatomical structural failures, such as the spring ligament complex and deltoid ligament complex, thus resulting in abnormal radiographic measurements.^8^, ^30^-^32^ Similar results were also supported by Prvulović *et al.*, who found that flatfoot was the most prevalent foot deformity in athletes.^12^

Our findings indicate no correlation between the CIA on radiography and posterior tendon tears assessed using US. This may have occurred because a few samples showed tendon tears on US. While tendon tears rarely occur in athletes in the acute clinical setting, all our patients had mild ankle discomfort and a history of chronic low-impact medial foot injury. Prvulović 33, 34 The majority of athletes in this study had flatfoot deformities and PTT tenosynovitis. Only very few athletes (6.3%) have tears in the PTT. This finding has a different result compared to previous studies by Hsu, T.C., and Wang, who reported 43.75% tendon tears in their total sample.^20^

Based on the type of sport, our study found that gymnastics had the highest percentage of flatfoot and tenosynovitis, followed by wrestling and fencing. Similar results were reported in other studies.^12^, ^33^ Wrestlers have also been found to have a higher prevalence of flatfoot compared to others.^12^ High stress levels and repetitive activity cause this.^35^ The state of the foot greatly depends on the type of effort, weight load carried, and the type of surface on which training and competition are carried, which differs in each type of sport. These situations increase the burden on foot arches. Flatfoot in athletes affects key elements of stability, ankle sprain, strength, agility, and mobility.^2^, ^13^, ^36^ Hence, holistic and comprehensive examinations of high-risk sports are important to prevent PTTD and flatfoot from occurring and worsening. Furthermore, lower back pain may occur due to bilateral flexible flat feet.^37^ In this study, the athletes and their coaches were able to have a better understanding of the risk factors and types of sports that tend to worsen flatfoot deformity and PTT dysfunction so that they could maintain optimal training to strengthen the medial ankle tendon-ligament complex.

This study has some limitations, including the lack of variety and type of sports, and a disproportional sample size in each type of sport, which interferes with further data analysis. Additionally, there is an absence of data on Body Mass Index (BMI) as a risk factor for AAFD. Future studies with larger sample sizes in a specific sport should be conducted to determine further which sports are risk factors for PTTD. This study did not include spring and deltoid ligament complexes as variables contributing to AAFD formation.

## Conclusion

This study showed a negative correlation between PTTD by ultrasound and AAFD by CIA in professional athletes with medial ankle pain and focal pain long the PTT. The correlation between PTTD and AAFD via ultrasound and CIA and its possible pathophysiological mechanisms can be especially useful for precise diagnosis to support comprehensive management. These imaging measurements may help clinicians and radiologists consider AAFD when the patient has PTTD in athletes with medial ankle pain and pain along the PTT. A better understanding of PTTD and AAFD imaging will lead to more effective management and prompt treatment, as AAFD affects the performance of athletes and predisposes them to further complications.

## Figures and Tables

**Table 1:** Description of sample characteristics

Number of participants	112
Male (%)	42
Female (%)	70
Mean age (years) (range)	18 (15-20)
Type of sport	
Athletics (%)	14 (13)
Wrestling (%)	34 (30)
Handball (%)	20 (18)
Fencing (%)	26 (23)
Wushu (%)	14 (12)
Gymnastics (%)	4 (4)
Feiss line test	
Normal	12 (11)
1^st^ degree (%)	75 (67)
2^nd^ degree (%)	23 (20)
3^rd^ degree (%)	2 (2)
Calcaneal inclination	
Normal (%)	33 (30)
Flatfoot (%)	79 (70)
Ultrasound	
Hypoechoic fluid surrounding tendon (mean ± SD) (median)**	(2,57 ± 1,6) (2,8)
Tendon sheath size (mean ± SD) (median)**	(8,25 ± 1,46) (8,2)
Tendon Tear (mean ± SD) (median)	(0,013 ± 0,05) (0)
Radiograph	
Calcaneal inclination angle (mean ± SD) (median)	(17,2 ± 3,12) (16,6)

*Note*. ** Inter-rater reliability by interclass correlation coefficient is (r = 0.89; CI 0.83, 0.94) and (r = 0.87; CI 0.78, 0.92) respectively

**Table 2:** Ultrasound and Radiograph Result in Type of Sport

Type of sports	Ultrasound			Radiograph	
	Normal (%)	Tenosynovitis(%)	Tear (%)	Normal (%)	Flatfoot (%)
Athletics	6/14(43)	8/14 (57)	1/14 (7)	5/14 (36)	9/14 (64)
Wrestling	10/34(29)	24/34 (71)	2/34 (6)	10/34 (29)	24/34 (71)
Handball	10/20(50)	10/20 (50)	1/20 (5)	7/20 (35)	13/20 (65)
Fencing	8/26(31)	18/26 (69)	2/26 (8)	4/26 (15)	22/26 (84)
Wushu	9/14(64)	5/14 (36)	1/14 (7)	7/14 (50)	7/14 (50)
Gymnastics	0/4(0)	4/4 (100)	0/4 (0)	0/4 (0)	4/4 (100)

**Table 3:** Correlation between tendon sheath fluid, tendon sheath thickness, tendon tear with calcaneal inclination angle

Variables	Calcaneal inclination angle		
	p**	r**	95% CI **
Tendon sheath fluid	<0.001	- 0,921	- 0.945, - 0.885
Tendon sheath thickening	<0.001	- 0,892	- 0.926, - 0.845
Tendon tear	0.728	- 0.33	- 0.223, - 0.159

*Note.* ** statistical analysis using Pearson's correlation
